# Publish and Perish: The Dangers of Being Young and in a Hurry

**DOI:** 10.7759/cureus.4098

**Published:** 2019-02-19

**Authors:** James S Huntley

**Affiliations:** 1 Surgery, Sidra Medicine, Ar-Rayyan, QAT

**Keywords:** publications, supervisor, supervision, routine activity frame-work, research misconduct, salami-slicing, inappropriate authorship, data falsification, plagiarism

## Abstract

Publications in peer-reviewed journals are a key and official requirement for progression to a consultant surgeon post. Paradoxically, a stipulation that should enhance the importance of surgical research may, in fact, contribute to a pressure that is one of the causes of research misconduct. Consultant trainers can go some way to mitigating against this danger with appropriate teaching and an emphasis on the core values surrounding research ethics.

## Editorial

The experience of "research" is thought of as an important focus across training in most medical specialties. There is an intense pressure to publish, which runs counter to good science and may encourage bad practice. This pressure exists even within my own - the most practical specialty: orthopedics.

To qualify as an orthopedic consultant in the UK, a trainee must achieve a Certificate of Completion of Training, a goal demanding certain personalities be negotiated (trainers), hoops jumped (competencies attained), and barriers scaled (representative case numbers and the Fellowship exam). The stipulations from the Joint Committee on Surgical Training (JCST) [1] also include:

“Trainees must also complete two of the following: (1) Higher degree completed at any time (MSc, MPhil, MD, PhD). (2) Authorship in any position (including corporate or collaborative) of two PubMed-cited papers relevant to the specialty, not including case reports. (3) A minimum of two presentations at national or international meetings. (4) Evidence of recruiting ≥5 patients into a research ethics committee approved study or ≥10 patients into a multi-centre observational study.”

It is not hard to make a case for the two publications because, despite some protests to the contrary, some aspects of surgery are scientific. Trainees should have the innate drive to engage with, question, and advance some aspect of technique or practice. Also, writing a paper and getting it published is itself an indicator of volition and tenacity. Candidates for consultant posts are likely to have similar credentials, and publications can be a critical discriminator. So, two publications are necessary and more/better publications are "highly desirable."

I contend that across the broad scope of surgery, research and surgical science are not well taught. Of course, there are established units with well-defined projects, trials, research agendas, and expertise. There are other pockets of brilliance but these are often understated, undervalued, and hard for trainees to identify. For trainees, genuine opportunities for research may be sparse and hard to distinguish from "millstones" doled out by well-meaning consultants, who may have few good ideas or, worse, many bad ones. Projects can be started that peter out or go nowhere. Time can be wasted, leading to a pressure build-up later in training.

Careful genuine guidance is vital - for many projects, it is wise to choose more than one "supervisor." They can be chosen to bring different expertise. One might bring lab space, clinical relevance, and broad oversight; another might understand the day-to-day intricacies of laboratory experiments; a third might provide clinical experience and patient/sample access. Supervisors must do more than merely garnish their name at the top of the paper. They should be the guarantor, knowledgeable of the data and record-keeping, and analysis; they should teach and oversee trainees in the ethics and the obligations/process of research and publication. Supervisors should have been educated themselves or, perceiving a deficiency, had themselves educated in those aspects of science/medicine/surgery they wish to pursue.

Research malfeasance is a crime, so perhaps it's right to consider the criminology literature. Perpetrators of crime are often painted in terms of means, motive, and opportunity. A classic paper [2] applying the "routine activity framework" approach to crime (albeit that of "direct-contact predatory violations") couched determinacy in terms of a similar (not identical) three "minimal elements": (i) "a motivated offender with intent and ability," (ii) "suitable targets," and (iii) "absence of a capable guardian."

There is a danger that by imposing a pressure to publish (Joint Committee on Surgical Training (JCST) stipulations [1] for career progression: "motivated'), in an inadequately supervised system ("absence of a capable guardian"), young, inexperienced bright surgeons ("intent and ability") doing research (a "suitable target") have a temptation ("motivated") to cheat, to take short-cuts. Linus Pauling allegedly observed *“*most of the cheats in science are MD*s*” [3].

Cheating - research misconduct - comes in many forms, most prominently: salami-slicing, duplication, inappropriate authorship, data falsification, and plagiarism [3-4]. I have seen them all in my half-life (hopefully, only half) in science and surgery. In particular, as a reviewer, I have identified plagiarism; I have heard a senior academic clinician justify data manipulation: “We must not let the data get in the way of the message”; I have stumbled across my prior unit's data stolen for another unit’s work (without citation); I have seen names at the top of papers, with the owners of those names never having seen the paper, let alone the final version; I have seen data from a student appropriated for a senior author's solo publication; I was once subjected to a barrage of (rejected) psychological abuse about loyalties of collaboration: "We wanted to welcome you to the inner sanctum but you continue to associate with that (scientist's name). To come with our group, you must sever all ties with him."

My point is that malfeasance is (unfortunately) only too common in surgical research.

We must support our colleagues and trainees, at the same time as providing oversight; this should not be so different from what we do clinically. It is ironic that the level of supervisorial input for a research paper is so variable, compared to the on-the-job audit ("critical analysis of the quality of medical care") we have in the form of weekly pre- and postoperative radiograph review, on most orthopedic units. Support comes largely in the form of appropriate education, and, again, similarly to the clinical environment, this is partly through seniors being exemplars. The majority of retractions are due to misconduct rather than honest error [5]. The ethics of scientific research are not complicated [4], but they must be emphasized as core values, from the outset.

As a profession, we have some way to go in terms of professionalism. It should be an obligatory journey, and like many journeys, it is largely about preparation and planning (Table 1).

**Table 1:** Basic advice for research alongside surgical training "Do's and Don'ts" of surgical trainee research

	Items	Notes
“DO”	understand the recommendations for scientific and scholarly work	(ICJME, 2017)
“DO”	understand ethical conduct and process of ethical approval	this involves both official (regulated) and non-regulated concerns
“DO”	start young and early	time spent thinking before starting is recouped later
“DO”	read in breadth and depth	consider the 'adjacent possible' - i.e. what next?
“DO”	count	develop and retain your primary data
“DO”	write	precise contemporaneous notes
“DO”	identify supervisors	– proper and multiple
“DON’T”	cheat/fabricate/lie/duplicate/plagiarise	
